# Seropositivity to canine tick-borne pathogens in a population of sick dogs in Italy

**DOI:** 10.1186/s13071-021-04772-9

**Published:** 2021-06-02

**Authors:** Jairo Alfonso Mendoza-Roldan, Giovanni Benelli, Marcos Antonio Bezerra-Santos, Viet-Linh Nguyen, Giuseppe Conte, Roberta Iatta, Tommaso Furlanello, Domenico Otranto

**Affiliations:** 1 Department of Veterinary Medicine, University of Bari, Valenzano, 70010 Bari, Italy; 2 Department of Agriculture, Food and Environment, University of Pisa, Pisa, Italy; 3 San Marco Veterinary Clinic and Laboratory, Veggiano, Padova, Italy; 4 Department of Pathobiology, Faculty of Veterinary Science, Bu-Ali Sina University, Hamedan, Iran

**Keywords:** *Anaplasma phagocytophilum*, *Borrelia burgdorferi*, *Ehrlichia canis*, *Rickettsia conorii*, Serological test, Vector-borne diseases

## Abstract

**Background:**

Canine vector-borne diseases (CVBDs) associated to ticks are among the most important health issues affecting dogs. In Italy, *Ehrlichia canis*, *Anaplasma* spp., *Rickettsia conorii* and *Borrelia burgdorferi* (*s.l.*) have been studied in both healthy canine populations and those clinically ill with suspected CVBDs. However, little information is currently available on the overall prevalence and distribution of these pathogens in the country. The aim of this study was to assess the prevalence and distribution of tick-borne pathogens (TBPs) in clinically suspect dogs from three Italian macro areas during a 15-year period (2006–2020).

**Methods:**

A large dataset (*n* = 21,992) of serological test results for selected TBPs in three macro areas in Italy was analysed using a Chi-square test to evaluate the associations between the categorical factors (i.e. macro area, region, year, sex and age) and a standard logistic regression model (significance set at *P* = 0.05). Serological data were presented as annual and cumulative prevalence, and distribution maps of cumulative positive cases for TBPs were generated.

**Results:**

Of the tested serum samples, 86.9% originated from northern (43.9%) and central (43%) Italy. The majority of the tests was requested for the diagnosis of *E. canis* (47%; *n* = 10,334), followed by *Rickettsia* spp. (35.1%; *n* = 7725), *B. burgdorferi* (*s.l.*) (11.6%; *n* = 2560) and *Anaplasma* spp. (6.2%; *n* = 1373). The highest serological exposure was recorded for *B. burgdorferi* (*s.l.*) (83.5%), followed by *Rickettsia* spp. (64.9%), *Anaplasma* spp. (39.8%) and *E. canis* (28.7%). The highest number of cumulative cases of *Borrelia burgdorferi* (*s.l.*) was recorded in samples from Tuscany, central Italy. *Rickettsia* spp. was more prevalent in the south and on the islands, particularly in dogs on Sicily older than 6 years, whereas *Anaplasma* spp. was more prevalent in the north and *E. canis* more prevalent in the south and on the islands.

**Conclusions:**

The results of this study highlight the high seroprevalence and wide distribution of the four TBPs in dogs with clinically suspected CVBDs from the studied regions of Italy. The very high seroprevalence of *B. burgdorferi* (*s.l.*) exemplifies a limitation of this study, given the use of clinically suspect dogs and the possibility of cross-reactions when using serological tests. The present research provides updated and illustrative information on the seroprevalence and distribution of four key TBPs, and advocates for integrative control strategies for their prevention.

**Grapic abstract:**

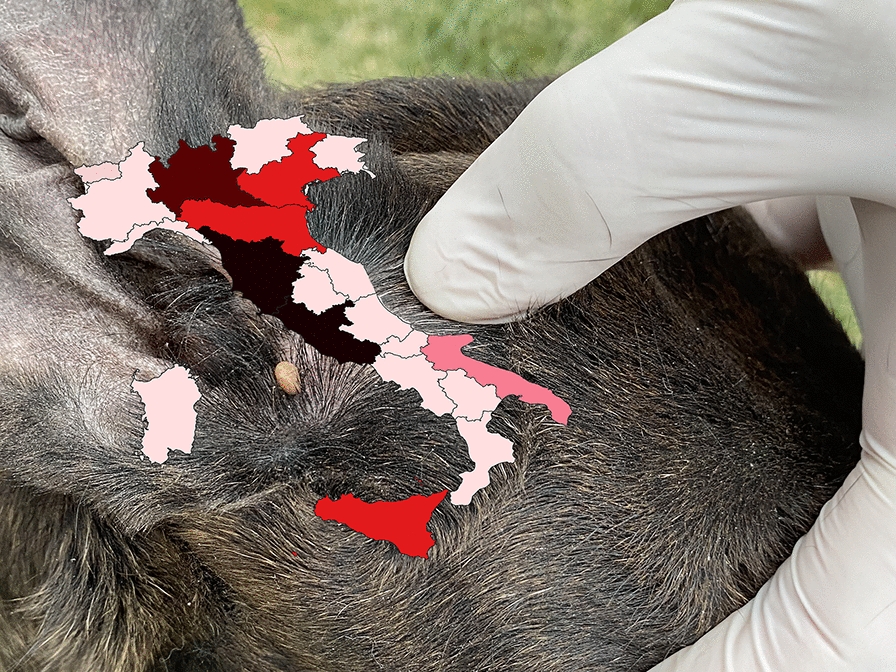

**Supplementary Information:**

The online version contains supplementary material available at 10.1186/s13071-021-04772-9.

## Background

Canine vector-borne diseases (CVBDs) are among the most important health issues affecting dogs worldwide [1]. Those transmitted by ticks are highly significant due to several factors related to the vector abundance, adaptation to different environments and climatic conditions [1, 2]. For example, the brown dog tick, *Rhipicephalus sanguineus* (*s.l.*), is considered to be a vector of several tick-borne pathogens (TBPs), such as *Ehrlichia canis*, *Anaplasma platys*, *Rickettsia conorii*, *Hepatozoon canis*, *Babesia vogeli* and *Cercopithifilaria* spp., in both rural and urban environments [3, 4], and as such to account for major veterinary and public health issues due to the disease burden caused by the transmission of TBPs in combination with scarce awareness of physicians and other health professionals [5]. Indeed, the prevalence of the pathogens transmitted by these arthropods in a specific geographical area is often associated with the abundance of competent tick vectors [6].

The most common tick-borne diseases of dogs are those caused by bacteria of the genera *Anaplasma*, *Ehrlichia*, *Rickettsia* and *Borrelia* [7]. *Ehrlichia canis*, the causative agent of canine monocytic ehrlichiosis, is a pathogen with a worldwide distribution that is endemic in several countries, mainly in tropical climates [8]. Similarly, infections by *Anaplasma* spp. (e.g. *Anaplasma phagocytophilum* and *A. platys*) in dogs have been recorded in many countries around the world [9–12]. In Italy, *E. canis* and *A. phagocytophilum* are common TBPs in domestic dogs, with reported seroprevalence as high as 46 and 38%, respectively [13–15]. *Rickettsia conorii* has been associated with acute febrile illness in dogs, with a seroprevalence ranging from 15.5 to 74% [14, 16]. Moreover, this bacterium poses a public health risk, as it is regarded as the etiological agent of Mediterranean spotted fever in humans, which is a serious disease characterized by maculo-papular rash, black eschar at the tick attachment site, high fever, flu-like symptoms and, in severe cases, major neurological symptoms and multi-organ failure [14]. Similarly, *Borrelia burgdorferi* (*s.l.*) is a zoonotic TBP reported in dogs from endemic regions, including many European countries such as Serbia, Italy and Croatia [11, 12, 17, 18]. However, seroprevalence of this bacterium in dogs has been reported to be lower (i.e. 0.3–5.4%) than that observed for Rickettsiales [12, 17] probably due to the sylvatic circulation of *B. burgdorferi* (*s.l.*) [19].

The distribution pattern of a pathogen is drastically affected by various factors, such as climate, travel history of the pet, importation of animals from endemic to non-endemic regions and vector ecology and abundance [20, 21]. Indeed, in the past few years CVBD emergence and re-emergence in Italy have shown different distribution patterns, as demonstrated for *Dirofilaria immitis* and *Leishmania infantum*, with the first becoming endemic in areas of central and southern Italy and the latter in northern regions [21]. However, for many TBPs there is a gap in scientific knowledge on their prevalence and distribution as much of the available information is from defined geographic areas, which does not provide the real picture of the epidemiology of such diseases at a nation-wide level.

In this scenario, the aim of the present study was to provide information on the distribution of major TBPs [i.e. *E. canis*, *Anaplasma* spp., *Rickettsia* spp., and *B. burgdorferi* (*s.l.*)] in clinically suspect dogs evaluated during a 15-year period (2006–2020) in a reference diagnostic centre in Italy.

## Methods

### Data collection

From June 2006 to May 2020 serum samples (*n* = 22,497) were collected from dogs suspected of having a TBD and sent to a diagnostic reference centre in Padova, Italy for the diagnosis of one or more of the following bacteria: *A. phagocytophilum*/*A. platys*, *B. burgdorferi* (*s.l.*), *E. canis* and *R. conorii*. Serum samples were tested using indirect immunofluorescence assays for the detection of immunoglobulin G (IgG) against *E. canis* antigens (sensitivity 92.3%, specificity 100%), *A. phagocytophilum* antigens (sensitivity 100%, specificity 96%) and *B. burgdorferi* (*s.l.*) antigens (sensitivity 90%, specificity 98.6%) using commercial kits (MegaFLUO® EHRLICHIA canis, MegaFLUO® ANAPLASMA, MegaFLUO® BORRELIA canis; all MEGACOR Diagnostik GmbH, Hoerbranz, Austria), according to the manufacturer's instructions. For the detection of IgG and IgM against *R. conorii* antigens (> 95% of sensitivity and specificity), commercial slides coated with antigen (Fuller Laboratories, Fullerton, CA, USA) and antibodies conjugated with fluorescein isothiocyanate anti-dog IgG (Sigma Aldrich, St. Louis, MO, USA) and anti-dog IgM (MEGACOR Diagnostik GmbH) were used according to the manufacturer’s instructions. Considering that *A. phagocytophilum* and *A. platys* are not distinguishable serologically, antibodies against *A. phagocytophilum* antigens were interpreted as anti-*Anaplasma* spp. antibodies. In the same way, antibodies against *R. conorii* antigens were interpreted as anti-*Rickettsia* spp. antibodies.

Sample origin was divided into three macro areas of Italy (i.e. north, central, and south/islands) according to the Italian geopolitical classification, as reported in [21]. Dogs were also categorized according to their age into the following groups: 0‒5, 6‒10 and > 10 years. Of the 22,497 serum samples collected, 505 were excluded from the analysis due to uncertainty regarding the sample origin. The results of the remaining 21,992 serodiagnosis tests along with data on origin, sex and age of tested animals were retrospectively reviewed and analysed.

### Statistical analysis

An exploratory evaluation of statistical associations between the categorical factors (i.e. macro area, region, year, sex and age) was carried out using the Chi-square test. Clustering in the final model was evaluated using the clinic attended as a random effect to compare the results from mixed-effects logistic regression modelling with standard logistic regression modelling [22]. Model fit was evaluated using the Hosmer–Lemeshow goodness-of-fit test statistic and the area under the receiver operating characteristic curve [22, 23]. Statistical significance was set at *P* = 0.05. The results from the logistic regression modelling are reported as an odds ratio and 95% confidence interval, which express the relative strength of association between the risk factor and the detected pathogen [i.e., *Anaplasma* spp., *B. burgdorferi* (*s.l.*), *E. canis*, and *Rickettsia* spp.]. Serological data were presented in terms of annual and cumulative seroprevalence; distribution maps of cumulative positive cases for different pathogens were generated using QGIS version 3.4.4-‘Madeira’.

## Results

The results of the serological tests performed in the three macro areas of Italy are reported in Table 1. Overall, 86.9% of the tested serum samples originated from northern and central Italy (43.9 and 43%, respectively); the remaining (13.1%) originated from southern Italy and the islands. Most of the tests were requested for the diagnosis of *E. canis* (47.0%; *n* = 10,334), followed by *Rickettsia* spp*.* (35.1%; *n* = 7725), *B. burgdorferi* (*s.l.*) (11.6%; *n* = 2560) and *Anaplasma* spp*.* (6.2%; *n* = 1373). The highest seroprevalence was for *B. burgdorferi* (*s.l.*) (83.5%), followed by *Rickettsia* spp. (64.9%), *Anaplasma* spp*.* (39.8%) and *E. canis* (28.7%).

**Table 1:** Results of serological diagnosis for pathogen infection in dogs in three macro areas of Italy from 2006 to 2020

Macro areas of Italy	*Anaplasma phagocytophilum*		*Borrelia burgdorferi*		*Ehrlichia canis*		*Rickettsia conorii*	
	*n*^a^	Positive test result^b^	*n*^a^	Positive test result^b^	*n*^a^	Positive test result^b^	*n*^a^	Positive test result^b^
North	774	321 (41.5%; 38.1‒45.1)	1529	1250 (81.8%; 79.7‒83.6)	4128	1166 (28.2%; 26.9‒29.6)	3231	1995 (61.7%; 60.1‒63.4)
Central	442	167 (37.8%; 33.4‒42.4)	945	818 (86.6%; 84.3‒88.6)	4361	1191 (27.3%; 26.1‒28.7)	3706	2459 (66.4%; 64.8‒67.9)
South and islands	157	58 (36.9%; 29.6‒44.9)	86	69 (80.2%; 70.4‒87.5)	1845	606 (32.8%; 30.7‒35.1)	788	559 (70.9%; 67.7‒74.1)
Total	1373	546 (39.8%; 37.2‒42.4)	2560	2137 (83.5%; 82‒84.9)	10,334	2963 (28.7%; 27.8‒29.6)	7725	5013 (64.9%; 63.8‒65.9)

^a^*n* = Number of tests

^b^Values are presented as a number, with the percentage and the 95% confidence interval (CI) given in parentheses

**Fig. 1:**
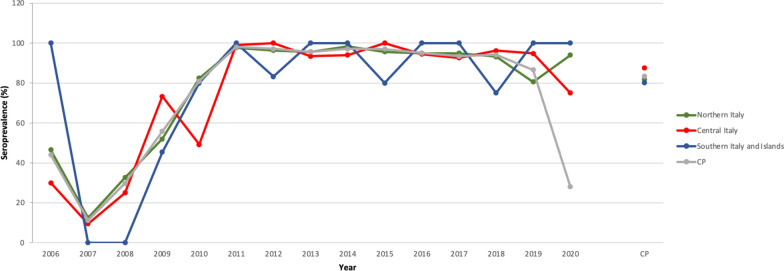
Pattern of mean annual seroprevalence of *Borrelia burgdorferi* (*s.l.*) in the three main macro areas of Italy. The cumulative prevalence (*CP*) is also shown

**Fig. 2:**
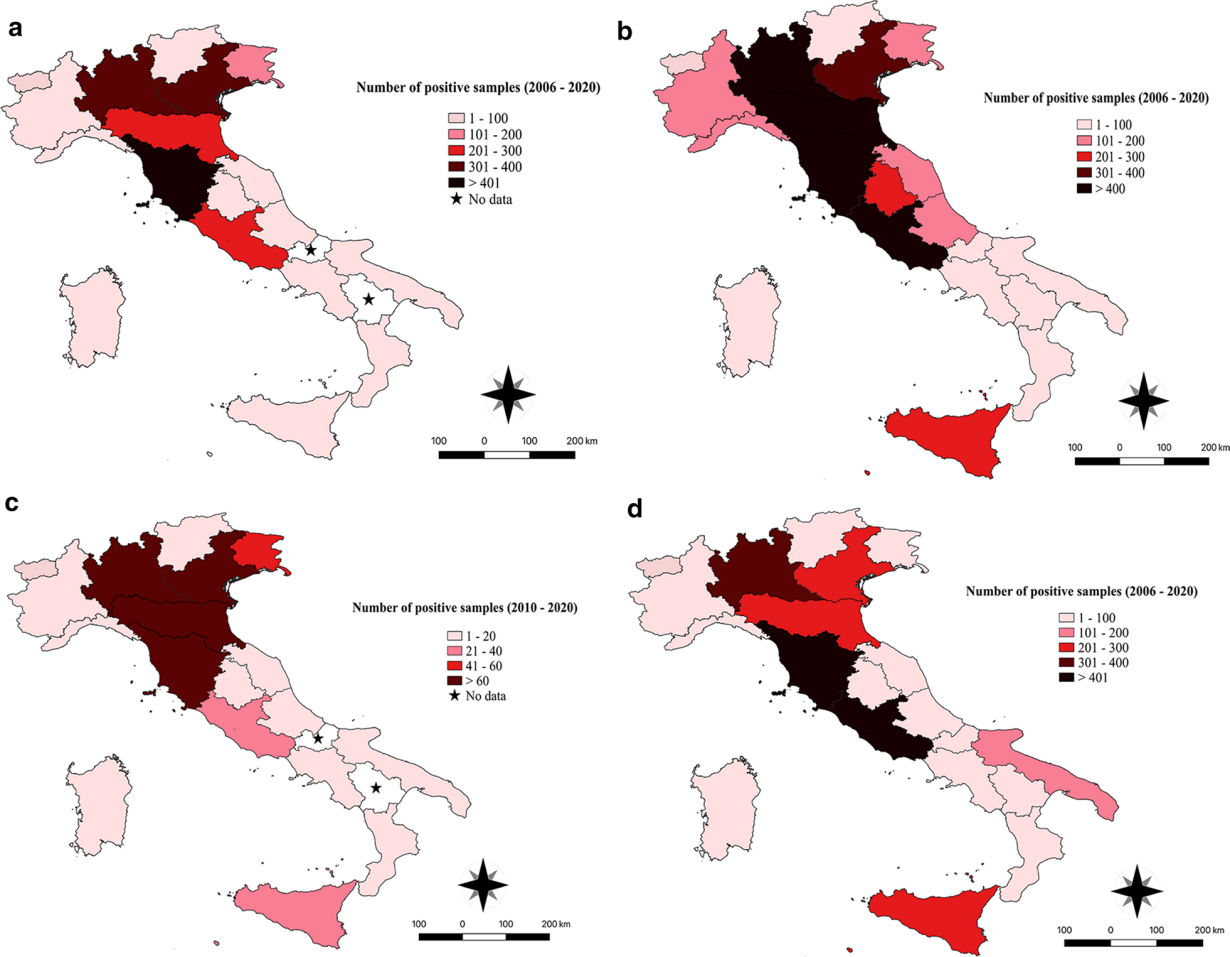
Distribution map of number of cases per region of Italy for *Borrelia burgdorferi* (*s.l.*) (**a**), *Rickettsia* spp. (**b**), *Anaplasma* spp. (**c**) and *Ehrlichia canis* (**d**)

The results of tests requested for the diagnosis of *B. burgdorferi* (*s.l.*) exposure (*n* = 2560) revealed an extremely high seropositivity among screened dogs in the three macro areas of Italy (Table 1), with the highest cumulative seroprevalence recorded in dogs from central Italy (*P* = 0.004) (Fig. 1; Additional file 1: Table S1). The highest cumulative number of dogs seropositive for *B. burgdorferi* (*s.l.*) was recorded in dogs from the Tuscany region, central Italy (Fig. 2a); however, no significant differences were noted among regions (Additional file 1: Table S1).

**Fig. 3:**
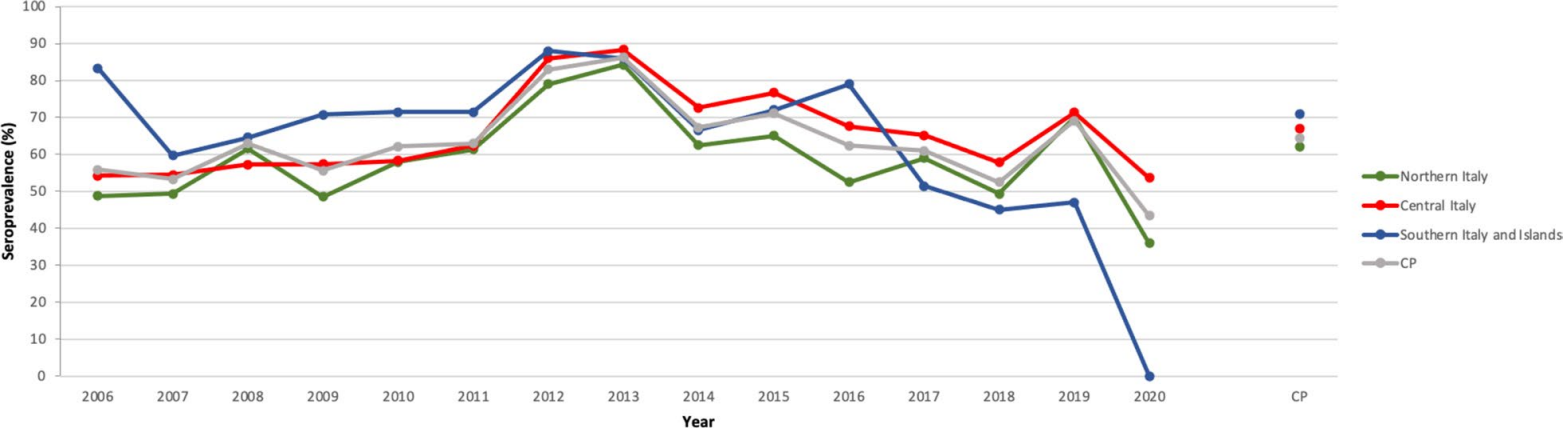
Pattern of mean annual seroprevalence of *Rickettsia* spp. in the three main macro areas of Italy studied. The CP is also shown

Dogs which tested positive for *B. burgdorferi* (*s.l.*) were more frequently observed in the 2010‒2015 and 2016‒2020 period than in the 2006‒2009 period (*P* < 0.001) (Additional file 1: Table S2). No significant difference was observed amongst dogs seropositive for *B. burgdorferi* (*s.l.*) regarding their sex, age and regional distribution (*P* > 0.05) (Additional file 1: Table S1). For tests requested for assessing *R. conorii* exposure (*n* = 7725), a significant difference among the macro areas was noted (Additional file 1: Tables S3, S4), with the highest seroprevalence recorded in dog populations originating from southern Italy and the islands and the lowest among dog populations from northern Italy (*P* < 0.001) (Table 1; Fig. 3). *Rickettsia* spp. seroprevalence varied significantly by region (*P* < 0.001), age (*P* < 0.001) and year (*P* < 0.001) (Additional file 1: Tables S5, S6 and S7, respectively), with the highest seroprevalence recorded for Sicily, Lazio, Tuscany, Emilia-Romagna, Friuli-Venezia Giulia and Lombardy (Fig. 2b) among dogs older than 6 years. During the period 2010‒2015, the number of dogs exposed was higher than those tested in the 2006‒2009 and 2016‒2020 periods. Dog sex was the only factor found not to be statistically different (Additional file 1: Table S3).

**Fig. 4:**
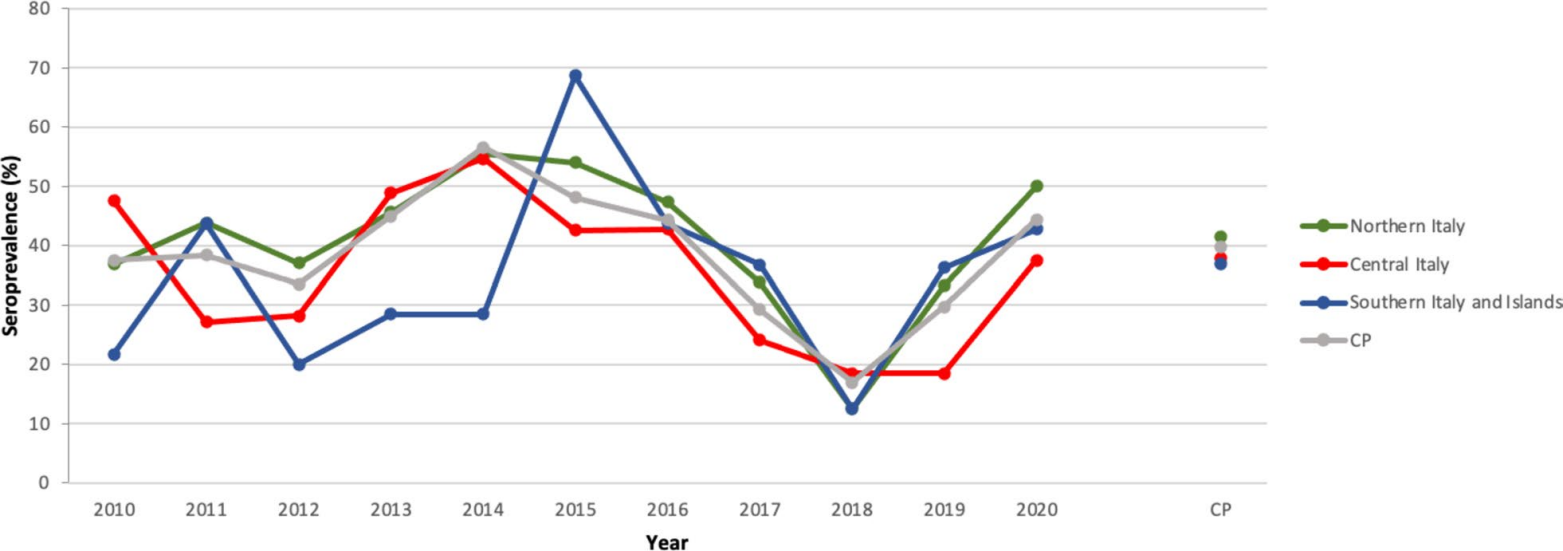
Pattern of mean annual seroprevalence of *Anaplasma* spp. in the three main macro areas of Italy. The CP is also shown

The analysis of tests requested for the diagnosis of *Anaplasma* spp*.* exposure (*n* = 1373) showed significant differences (*P* < 0.05) in the following factors: region and year of evaluation. Indeed, exposed dogs were more frequently observed in the 2016‒2020 period (23%) than in the 2010‒2015 (8%) period (*P* < 0.001) (Table 1; Fig. 4; Additional file 1: Table S8). Significant differences were noted among regions (Additional file 1: Table S9); those regions with the lowest percentage of dogs exposed to *Anaplasma* spp*.* were Calabria, Sardinia, Abruzzo, Marche, Umbria (14‒23%) (Fig. 2c), while the other regions showed a more balanced distribution between exposed and non-exposed dogs, with the exception of Trentino Alto-Adige, which was the only region where the percentage of affected dogs was higher than that of non-affected dogs.

**Fig. 5:**
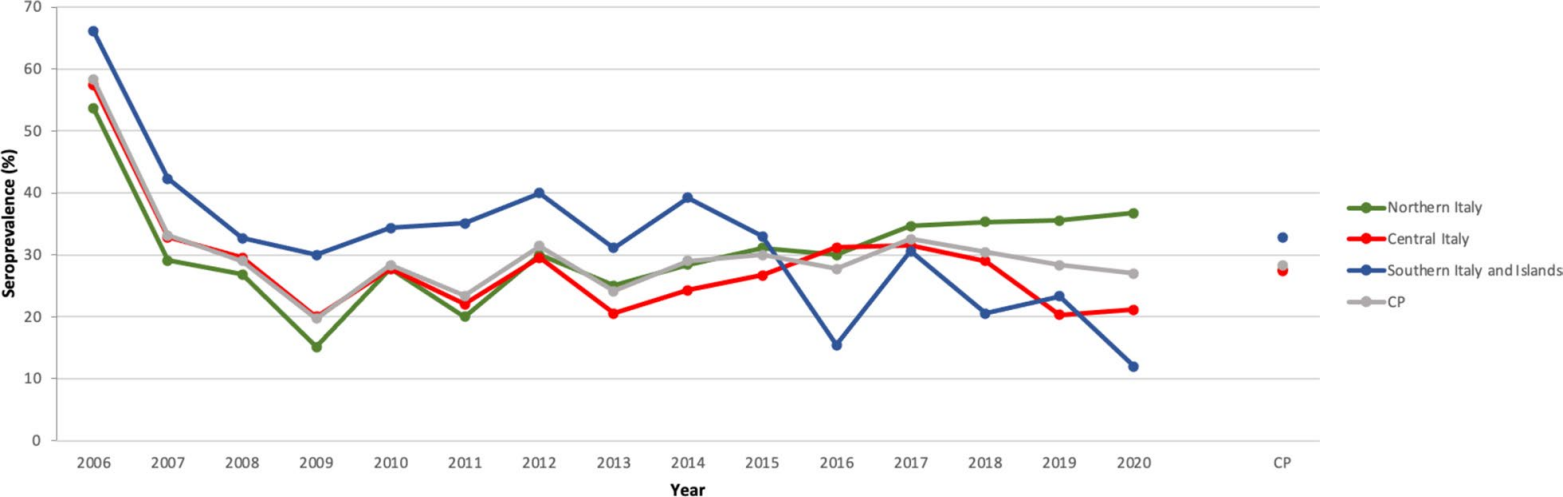
Pattern of mean annual seroprevalence of *Ehrlichia canis* in the three main macro areas of Italy. The CP is also shown

A significant difference in macro areas, regional distribution and year of evaluation was observed in dogs examined for *E. canis* (*n* = 10,334) (Additional file 1: Table S10). The seroprevalence was higher among dogs from regions in southern Italy (e.g. Apulia and Calabria) and on Sicily than among those from regions in central and northern Italy (Table 1; Fig. 5). Conversely, the highest cumulative number of cases was reported from northern (i.e. Lombardy) and central (i.e. Lazio and Tuscany) Italy and from Sicily (Fig. 2d) (Additional file 1: Table S11). The results of the samples tested in 2016‒2020 period revealed a higher infection rate compared to the previous years (Additional file 1: Table S12).

## Discussion

Data obtained from a large sample of dogs suspected of being clinically ill during a 15-year period showed the seroprevalence and distribution of four key TBPs in Italy, as well as an increasing trend of cumulative seroprevalence and number of cases throughout the peninsula, with a high seroprevalence in the northern and central areas of Italy. The fact that most of the tests requested originated from northern and central regions could limit the overall interpretation of the results given that southern regions and the islands were underrepresented. Overall, the seroprevalence of *Rickettsia* spp. (64.9%), *Anaplasma* spp. (39.8%) and *E. canis* (28.7%) were similar to that reported in previous studies in Italy [13–16]. In contrast, *B. burgdorferi* (*s.l.*) seroprevalence throughout Italy (83.5%) was higher than that previously reported from central (1.4%) and southern (5.4%-7.8%) regions [17, 24, 25]. These results need to be assessed cautiously given the study limitations (e.g. unknown clinical and travel history of clinically suspect dogs and possibility of cross-reactions with other spirochetes) that could hinder their proper interpretation. Nonetheless, the present study shows for the first time the exposure of dogs to canine borreliosis in the northern regions, although the low number of tests requested for some regions prevents a clear epidemiological picture of the distribution of this TBP. In addition, serological arrays, in general, are not capable of distinguishing and differentiating between *Borrelia* spp. since cross-reaction impedes specific identification of the borrelial causative agent [26]. Thus, serological studies should be conducted with molecular identification, and more epidemiological surveys are needed to determine the composition of borrelial species in dogs from Italy [26]. Previous molecular studies have shown a prevalence of up to 60% of animals manifesting clinical signs of borreliosis [27]; thus a high seroprevalence of animals suspected for unspecific symptoms of any given TBD may be expected. However, the high seroprevalence for *B. burgdorferi* (*s.l.*) in clinically suspect dogs may represent an overlap of specific clinical signs of canine borreliosis with an increased awareness of clinicians to the disease in canine and human populations. Indeed, recent estimates of borrelial infection and incidence suggest that prevalence is much higher than published data from official health authorities would indicate, with an underestimation of real clinical cases in humans and companion animals [28]. In particular, the high seroprevalence of canine borreliosis in the northern and central regions of Italy may be due to the distribution of the main vector, *Ixodes ricinus*, in these regions [29, 30]. In support of this, the highest cumulative prevalence (86.6%) and number of cases (i.e. 539) were observed in the central region (i.e. Tuscany) where *I. ricinus* is prevalent [31]. In addition, the seroprevalence for *B. burgdorferi* (*s.l.*) recorded herein in Italy (83.5%) is much higher than that recorded in dogs from Switzerland (i.e. 57.5%) [32].

A previous study assessing TBPs in diseased dogs from Italy recorded a similar seroprevalence of *Rickettsia* spp. for the three macro areas (e.g. 68% for the central region) [15]. Accordingly, in the southern regions and on the islands, the prevalence of seropositive animals (71%) was similar to that reported in [13]. Moreover, the distribution of *Rickettsia* spp. has changed in the last decade, from mainly occurring in southern Italy [16, 33] to occurring in the northern/central regions. *Rickettsia conorii* can infect dogs, causing fever and other tick-borne unspecific symptoms, and the main tick vector [i.e. *R. sanguineus* (*s.l.*)] is prevalent throughout Italy [33]. Although, it is still unclear the role dogs play as reservoirs of *Rickettsia* spp*.*, they may transmit the bacteria to ticks feeding on them [34]. Dogs older than 6 years had a higher rate of infection, suggesting seroconversion, chronic onset of the disease and increased exposure to the vector [14, 35]. Furthermore, other *Rickettsia* spp. from the spotted fever group occur in Italy, potentially leading to serological cross-reactivity in exposed animals [36].

Overall, the seroprevalence of *Anaplasma* spp. (41.5%) was higher in the northern regions than in the southern regions of Italy, while the opposite was true for *E. canis* (32.8%). Other studies in the same geographical areas as the present study reported different results, with the seroprevalence of *Anaplasma* spp. higher in the central area (i.e. 46%) and lower in the northern area (i.e. 10%) [15], while other serological surveys recorded a lower prevalence for both pathogens (i.e. 3.31–4.7% for *Anaplasma* spp. and 7–16.2% for *E. canis*) in the central regions [24, 37]. The high seroprevalence for *Anaplasma* spp. in the northern area also differs from previous studies (i.e. 3.3–4.7%), suggesting a higher exposure to the tick vectors in northern and central regions [38, 39].

As tested sera from the present study were from clinically suspect dogs, a cross-reactivity for both bacteria cannot be ruled out since *A. platys* may cross-react with *A. phagocytophilum* and, when low antibody levels are present, with *E. canis.* Given that information on the origin and travel history of the tested dogs was not available, cross-reactivity with *Ehrlichia* spp. not currently known to be present in Italy cannot be excluded, although unlikely considering that *E. canis* is the only *Ehrlichia* species isolated from dogs in Europe [37, 40]. Moreover, *E. canis* seroprevalence in the southern regions, mainly on Sicily, was similar to that previously reported in the same geographical area (i.e. 29.6–46%) [13, 35], possibly due to the wide circulation of *R. sanguineus* (*s.l.*) throughout the Mediterranean basin [33, 41].

Since these pathogens [i.e. *B. burgdorferi* (*s.l.*), *Rickettsia* spp., *Anaplasma* spp., and *Ehrlichia* spp.] may cause diseases with overlapping clinical manifestations, identifying the causative agent of any suspected CVBDs by serological means, could turn to be difficult. In fact, lower molecular prevalence is usually detected in surveys that correlate molecular and serological findings, even in sick dogs [15, 34], due to the transient bacteraemia that appears soon after the infective tick bites [42].

## Conclusions

Assessment in this study of a large dataset for the most common TBPs of dogs [i.e. *E. canis*, *Anaplasma* spp., *Rickettsia* spp*.*, and *B. burgdorferi* (*s.l.*)] suggest the endemic and wide distribution of these TBPs in the Italian peninsula and on the islands. Overall, a high prevalence of most of these pathogens was expected given that the analysed sera were obtained from dogs suspected of being clinically ill. However, the high seroprevalence of *B. burgdorferi* (*s.l.*) exemplifies a limitation of this study, given the use of clinically suspect dogs and the possibility of cross-reactions when using serological tests. This study is the first serological survey to analyse data over a 15 year-period. This analysis enabled us to generate an updated picture of prevalence and distribution of these TBPs in Italy, which can be used for risk assessment per region, year, sex and age of the animals. Furthermore, this study provides important information on the prevalence and distribution of these four TBPs, which can be useful for veterinarians and public health officials, who should be aware of the nation-wide distribution of these bacteria and their zoonotic potential. Indeed, integrative molecular and serological surveys are advocated for a better epidemiological surveillance toward the assessment of proper preventative control measures.

## Supplementary Information

**Additional file 1: Table S1.** Characteristics of a cohort of 2560 dogs tested for *Borrelia burgdorferi* (*s.l.*); data are expressed as the number of dogs (% on the total of each category). The frequency of categories for each variable was compared across affected and non-affected status by a *χ*^2^ test. *P* < 0.05 was considered to be significant. **Table S2.** Significant explanatory variable “year” associated with the infection by *Borrelia burgdorferi* (*s.l.*), based on multivariate logistic regression; the numbers represent odds ratio of category on the row* vs* category on the column. **Table S3.** Characteristics of a cohort of 7725 dogs tested for *Rickettsia* spp.; data are expressed as number of subjects (% of the total of each category). The frequency of categories for each variable was compared across affected and non-affected status by a *χ*^2^ test. *P* < 0.05 was considered to be significant. **Table S4.** Significant explanatory variable “macro area” associated with the infection by *Rickettsia* spp., based on multivariate logistic regression; the numbers represent odds ratio of category on the row* vs* category on the column. **Table S5.** Significant explanatory variable “Italian region” associated with the infection by *Rickettsia* spp., based on multivariate logistic regression; the numbers represent odds ratio of category on the row* vs* category on the column. **Table S6.** Significant explanatory variable “dog age” associated with the infection by *Rickettsia* spp.*,* based on multivariate logistic regression; the numbers represent odds ratio of category on the row* vs* category on the column. **Table S7.** Significant explanatory variable “year” associated with the infection by *Rickettsia* spp., based on multivariate logistic regression; the numbers represent odds ratio of category on the row* vs* category on the column. **Table S8.** Characteristics of a cohort of 1373 dogs tested for *Anaplasma* spp.; data are expressed as the number of dogs (% on the total of each category). The frequency of categories for each variable was compared across affected and non-affected status by a *χ*^2^ test. *P* < 0.05 was considered to besignificant. **Table S9.** Significant explanatory variable “Italian region” associated with the infection by *Anaplasma* spp., based on multivariate logistic regression; the numbers represent odds ratio of category on the row* vs* category on the column. **Table S10.** Characteristics of a cohort of 10,334 dogs tested for *Ehrlichia canis*; data are expressed as number of dogs (% on the total of each category). The frequency of categories for each variable was compared across affected and non-affected status by a *χ*^2^ test. *P* < 0.05 was considered to be significant. **Table S11.** Significant explanatory variable “Italian region” associated with the infection by *Ehrlichia canis*, based on multivariate logistic regression; the numbers represent odds ratio of category on the row* vs* category on the column. **Table S12.** Significant explanatory variable “year” associated with the infection by *Ehrlichia canis*, based on multivariate logistic regression; the numbers represent odds ratio of category on the row* vs* category on the column.

## Data Availability

Data supporting the conclusions of this article are included within the article and its additional files. The raw datasets used and analysed during the current study are available from the corresponding author upon reasonable request.

## Abbreviations

CVBDs: Canine vector-borne diseases
TBPs: Tick-borne pathogens
IgG: Immunoglobulin G
CP: Cumulative prevalence
