# Supplementary material for: Seropositivity to canine tick-borne pathogens in a population of sick dogs in Italy
Source: Parasit Vectors. 2021 Jun 2;14:292. doi: 10.1186/s13071-021-04772-9 (PMC8171035; doi:10.1186/s13071-021-04772-9)
Supplement: Supplementary file 1 — Additional file 1: Table S1. Characteristics of a cohort of 2560 dogs tested for Borrelia burgdorferi (s.l.); data are expressed as the number of dogs (% on the total of each category). The frequency of categories for each variable was compared across affected and non-affected status by a χ2 test. P < 0.05 was considered to be significant. Table S2. Significant explanatory variable “year” associated with the infection by Borrelia burgdorferi (s.l.), based on multivariate logistic regression; the numbers represent odds ratio of category on the row vs category on the column. Table S3. Characteristics of a cohort of 7725 dogs tested for Rickettsia spp.; data are expressed as number of subjects (% of the total of each category). The frequency of categories for each variable was compared across affected and non-affected status by a χ2 test. P < 0.05 was considered to be significant. Table S4. Significant explanatory variable “macro area” associated with the infection by Rickettsia spp., based on multivariate logistic regression; the numbers represent odds ratio of category on the row vs category on the column. Table S5. Significant explanatory variable “Italian region” associated with the infection by Rickettsia spp., based on multivariate logistic regression; the numbers represent odds ratio of category on the row vs category on the column. Table S6. Significant explanatory variable “dog age” associated with the infection by Rickettsia spp., based on multivariate logistic regression; the numbers represent odds ratio of category on the row vs category on the column. Table S7. Significant explanatory variable “year” associated with the infection by Rickettsia spp., based on multivariate logistic regression; the numbers represent odds ratio of category on the row vs category on the column. Table S8. Characteristics of a cohort of 1373 dogs tested for Anaplasma spp.; data are expressed as the number of dogs (% on the total of each category). The frequency of categories for [file 13071_2021_4772_MOESM1_ESM.docx]

**Additional file 1**

***Borrelia burgdorferi***

Table S1. Characteristics of a cohort of 2560 dogs infected by *B. burgdorferi*; data are expressed as n° of subjects (% on the total of each category). The frequency of categories for each variable was compared across affected and non-affected status by a *χ^2^* test, *P*<0.05 was considered significant.

| Variable | Category |  | No | Yes | *P*-value |
| --- | --- | --- | --- | --- | --- |
| Macro area | Northern Italy |  | 277 (18%) | 1252 (82%) | 0.004 |
|  | Central Italy |  | 127 (13%) | 818 (87%) |  |
|  | Southern Italy |  | 17 (20%) | 69 (80%) |  |
| Sex | Female |  | 195 (15%) | 1058 (85%) | 0.210 |
|  | Male |  | 228 (17%) | 1079 (83%) |  |
| Age | 0-5 years |  | 227 (17%) | 1133 (83%) | 0.740 |
|  | 6-10 years |  | 145 (16%) | 760 (84%) |  |
|  | >10 years |  | 51 (17%) | 244 (83%) |  |
| Region | Apulia |  | 2 (13%) | 14 (87%) | 0.308 |
|  | Calabria |  | 1 (8%) | 11 (82%) |  |
|  | Campania |  | 1 (25%) | 3 (75%) |  |
|  | Sardinia |  | 2 (11%) | 17 (89%) |  |
|  | Sicily |  | 10 (29%) | 25 (71%) |  |
|  | Abruzzo |  | 2 (33%) | 4 (67%) |  |
|  | Lazio |  | 52 (20%) | 206 (80%) |  |
|  | Marche |  | 20 (25%) | 63 (75%) |  |
|  | Tuscany |  | 52 (9%) | 539 (81%) |  |
|  | Umbria |  | 1 (15%) | 6 (85%) |  |
|  | Emilia-Romagna |  | 53 (16%) | 279 (84%) |  |
|  | Friuli-Venezia Giulia |  | 42 (20%) | 165 (80%) |  |
|  | Liguria |  | 23 (25%) | 67 (75%) |  |
|  | Lombardy |  | 74 (18%) | 338 (82%) |  |
|  | Piedmont |  | 29 (26%) | 83 (74%) |  |
|  | Trentino Alto-Adige |  | 11 (48%) | 12 (52%) |  |
|  | Aosta Valley |  | 0 (0%) | 2 (100%) |  |
|  | Veneto |  | 47 (13%) | 304 (87%) |  |
| Year | 2006-2009 |  | 276 (60%) | 180 (40%) | <0.001 |
|  | 2010-2015 |  | 87 (7%) | 1198 (83%) |  |
|  | 2016-2020 |  | 59 (7%) | 760 (83%) |  |

Table S2: Significant explanatory variable “year” associated with the infection by *B. burgdorferi,* based on multivariate logistic regression; the numbers represent odds ratio of category on the row versus category on the column.

|  | 2006-2009 | 2010-2015 | 2016-2020 |
| --- | --- | --- | --- |
| 2006-2009 | - |  |  |
| 2010-2015 | 0.05*** | - |  |
| 2016-2020 | 0.06*** | 1.16 | - |

*** = *P* < 0.001

***Rickettsia conorii***

Table S3. Characteristics of a cohort of 7725 dogs infected by *R. conorii*; data are expressed as n° of subjects (% on the total of each category). The frequency of categories for each variable was compared across affected and non-affected status by a *χ^2^* test, *P*<0.05 was considered significant.

| Variable | Category |  | No | Yes | *P*-value |
| --- | --- | --- | --- | --- | --- |
| Macro area | Northern Italy |  | 1236 (38%) | 1995 (62%) | <0.001 |
|  | Central Italy |  | 1247 (33%) | 2459 (67%) |  |
|  | Southern Italy |  | 229 (29%) | 559 (71%) |  |
| Sex | Female |  | 1307 (36%) | 2300 (64%) | 0.07 |
|  | Male |  | 1405 (34%) | 2713 (66%) |  |
| Age | 0-5 years |  | 1385 (37%) | 2332 (63%) | <0.001 |
|  | 6-10 years |  | 997 (33%) | 2013 (67%) |  |
|  | >10 years |  | 330 (33%) | 668 (67%) |  |
| Region | Apulia |  | 25 (32%) | 52 (68%) | <0.001 |
|  | Basilicata |  | 3 (7%) | 33 (93%) |  |
|  | Calabria |  | 33 (27%) | 88 (73%) |  |
|  | Campania |  | 14 (31%) | 30 (69%) |  |
|  | Molise |  | 14 (26%) | 39 (74%) |  |
|  | Sardinia |  | 21 (28%) | 53 (72%) |  |
|  | Sicily |  | 119 (32%) | 264 (68%) |  |
|  | Abruzzo |  | 43 (28%) | 110 (72%) |  |
|  | Lazio |  | 686 (39%) | 1050 (61%) |  |
|  | Marche |  | 166 (40%) | 260 (60%) |  |
|  | Tuscany |  | 305 (25%) | 921 (75%) |  |
|  | Umbria |  | 47 (29%) | 118 (71%) |  |
|  | Emilia-Romagna |  | 294 (36%) | 521 (64%) |  |
|  | Friuli-Venezia Giulia |  | 99 (38%) | 158 (62%) |  |
|  | Liguria |  | 49 (26%) | 133 (74%) |  |
|  | Lombardy |  | 400 (39%) | 641 (61%) |  |
|  | Piedmont |  | 117 (47%) | 131 (53%) |  |
|  | Trentino Alto-Adige |  | 15 (37%) | 28 (63%) |  |
|  | Aosta Valley |  | 0 (0%) | 7 (100%9 |  |
|  | Veneto |  | 262 (41%) | 376 (59%) |  |
| Year | 2006-2009 |  | 1022 (42%) | 1403 (58%) | <0.001 |
|  | 2010-2015 |  | 1090 (29%) | 2703 (71%) |  |
|  | 2016-2020 |  | 600 (40%) | 907 (60%) |  |

Table S4: Significant explanatory variable “macro area” associated with the infection by *R. conorii,* based on multivariate logistic regression; the numbers represent odds ratio of category on the row versus category on the column.

|  | Northern Italy | Central Italy | Southern Italy |
| --- | --- | --- | --- |
| Northern Italy | - |  |  |
| Central Italy | 0.82*** | - |  |
| Southern Italy | 0.67*** | 0.82* | - |

* = *P* < 0.05; *** = *P* < 0.001

Table S5: Significant explanatory variable “Italian region” associated with the infection by *R. conorii,* based on multivariate logistic regression; the numbers represent odds ratio of category on the row versus category on the column.

|  | Apulia | Basilicata | Calabria | Campania | Molise | Sardinia | Sicily | Abruzzo | Lazio | Marche | Tuscany | Umbria | Emilia-Romagna | Friuli-Venezia Giulia | Liguria | Lombardy | Piedmont | Trentino Alto-Adige | Vale d'Aosta | Veneto |
| --- | --- | --- | --- | --- | --- | --- | --- | --- | --- | --- | --- | --- | --- | --- | --- | --- | --- | --- | --- | --- |
| Apulia | - |  |  |  |  |  |  |  |  |  |  |  |  |  |  |  |  |  |  |  |
| Basilicata | 0.21* | - |  |  |  |  |  |  |  |  |  |  |  |  |  |  |  |  |  |  |
| Calabria | 0.76 | 3.68* | - |  |  |  |  |  |  |  |  |  |  |  |  |  |  |  |  |  |
| Campania | 1.00 | 4.82* | 1.31 | - |  |  |  |  |  |  |  |  |  |  |  |  |  |  |  |  |
| Molise | 0.71 | 3.43* | 0.93 | 0.71 | - |  |  |  |  |  |  |  |  |  |  |  |  |  |  |  |
| Sardinia | 0.73 | 3.55* | 0.96 | 0.74 | 1.03 | - |  |  |  |  |  |  |  |  |  |  |  |  |  |  |
| Sicily | 0.79 | 3.82* | 1.04 | 0.79 | 1.11 | 1.08 | - |  |  |  |  |  |  |  |  |  |  |  |  |  |
| Abruzzo | 0.66 | 3.18* | 0.86 | 0.66 | 0.93 | 0.90 | 0.83 | - |  |  |  |  |  |  |  |  |  |  |  |  |
| Lazio | 1.16 | 5.63** | 1.53* | 1.17 | 1.64 | 1.59 | 1.47 | 1.77** | - |  |  |  |  |  |  |  |  |  |  |  |
| Marche | 1.17 | 5.68** | 1.54* | 1.18 | 1.65 | 1.60 | 1.49** | 1.79** | 1.01 | - |  |  |  |  |  |  |  |  |  |  |
| Tuscany | 0.63 | 3.05* | 0.83 | 0.63 | 0.89 | 0.86 | 0.80 | 0.96 | 0.54*** | 0.54*** | - |  |  |  |  |  |  |  |  |  |
| Umbria | 0.81 | 3.91* | 1.06 | 0.81 | 1.14 | 1.10 | 1.02 | 1.23 | 0.70* | 0.69* | 1.28 | - |  |  |  |  |  |  |  |  |
| Emilia-Romagna | 1.09 | 5.27** | 1.43 | 1.09 | 1.53 | 1.48 | 1.38 | 1.66* | 0.94 | 0.93 | 1.73*** | 1.35 | - |  |  |  |  |  |  |  |
| Friuli-Venezia Giulia | 1.17 | 5.67** | 1.54 | 1.18 | 1.65 | 1.60 | 1.49 | 1.79** | 1.01 | 1.00 | 1.86*** | 1.45 | 1.08 | - |  |  |  |  |  |  |
| Liguria | 0.66 | 3.17* | 0.86 | 0.66 | 0.92 | 0.89 | 0.83 | 1.00 | 0.56*** | 0.56** | 1.04 | 0.81 | 0.60 | 0.56* | - |  |  |  |  |  |
| Lombardy | 1.22 | 5.88*** | 1.60* | 1.22 | 1.71 | 1.66 | 1.54** | 1.85*** | 1.05 | 1.04 | 1.93*** | 1.50* | 1.12 | 1.04 | 1.85*** | - |  |  |  |  |
| Piedmont | 1.60 | 7.76*** | 2.11** | 1.61 | 2.26* | 2.19** | 2.03*** | 2.44*** | 1.38* | 1.37 | 2.55*** | 1.98** | 1.47** | 1.37 | 2.44*** | 1.32 | - |  |  |  |
| Trentino Alto-Adige | 0.98 | 4.73*** | 1.28 | 0.98 | 1.38 | 1.33 | 1.24 | 1.49 | 0.84 | 0.83 | 1.55 | 1.21 | 0.90 | 0.83 | 1.49 | 0.80 | 0.61 | - |  |  |
| Aosta Valley | ne | ne | ne | ne | ne | ne | ne | ne | ne | ne | ne | ne | ne | ne | ne | ne | ne | ne | - |  |
| Veneto | 1.30 | 6.28** | 1.71* | 1.30 | 1.83* | 1.77* | 1.64*** | 1.98*** | 1.12 | 1.11 | 2.06*** | 1.61* | 1.19 | 1.11 | 1.98*** | 1.07 | 0.81 | 1.33 | ne | - |

* = *P* < 0.05; ** = *P* < 0.01; *** = *P* < 0.001; ne = not estimable

Table S6: Significant explanatory variable “dog age” associated with the infection by *R. conorii,* based on multivariate logistic regression; the numbers represent odds ratio of category on the row versus category on the column.

|  | 0-5 | 6-10 | >11 |
| --- | --- | --- | --- |
| 0-5 | - |  |  |
| 6-10 | 0.83*** | - |  |
| >10 | 0.83* | 1.00 | - |

* = *P* < 0.05; *** = *P* < 0.001

Table S7: Significant explanatory variable “year” associated with the infection by *R. conorii,* based on multivariate logistic regression; the numbers represent odds ratio of category on the row versus category on the column.

|  | 2006-2009 | 2010-2015 | 2016-2020 |
| --- | --- | --- | --- |
| 2006-2009 | - |  |  |
| 2010-2015 | 0.56*** | - |  |
| 2016-2020 | 0.90 | 1.62*** | - |

*** = *P* < 0.001

***Anaplasma* *phagocytophilum***

Table S8. Characteristics of a cohort of 1373 dogs infected by *A.* *phagocytophilum*; data are expressed as n° of subjects (% on the total of each category). The frequency of categories for each variable was compared across affected and non-affected status by a *χ^2^* test, *P*<0.05 was considered significant.

| Variable | Category |  | No | Yes | *P*-value |
| --- | --- | --- | --- | --- | --- |
| Macro area | Northern Italy |  | 451 (58%) | 321 (42%) | 0.468 |
|  | Central Italy |  | 277 (62%) | 167 (38%) |  |
|  | Southern Italy |  | 99 (63%) | 58 (37%) |  |
| Sex | Female |  | 390 (58%) | 285 (42%) | 0.09 |
|  | Male |  | 437 (63%) | 261 (37%) |  |
| Age | 0-5 years |  | 426 (63%) | 255 (37%) | 0.277 |
|  | 6-10 years |  | 289 (58%) | 209 (42%) |  |
|  | >10 years |  | 112 (58%) | 82 (42%) |  |
| Region | Apulia |  | 22 (55%) | 18 (45%) | <0.001 |
|  | Calabria |  | 15 (83%) | 3 (17%) |  |
|  | Campania |  | 1 (50%) | 1 (50%) |  |
|  | Sardinia |  | 34 (77%) | 10 (23%) |  |
|  | Sicily |  | 27 (51%) | 26 (49%) |  |
|  | Abruzzo |  | 6 (86%) | 1 (14%) |  |
|  | Lazio |  | 63 (67%) | 31 (33%) |  |
|  | Marche |  | 21 (84%) | 4 (16%) |  |
|  | Tuscany |  | 162 (56%) | 127 (44%) |  |
|  | Umbria |  | 23 (85%) | 4 (15%) |  |
|  | Emilia-Romagna |  | 106 (56%) | 82 (44%) |  |
|  | Friuli-Venezia Giulia |  | 71 (58%) | 51 (42%) |  |
|  | Liguria |  | 26 (68%) | 12 (31%) |  |
|  | Lombardy |  | 107 (59%) | 75 (41%) |  |
|  | Piedmont |  | 18 (56%) | 14 (44%) |  |
|  | Trentino Alto-Adige |  | 9 (35%) | 17 (65%) |  |
|  | Aosta Valley |  | 1 (100%) | 0 (0%) |  |
|  | Veneto |  | 116 (63%) | 69 (37%) |  |
| Year | 2010-2015 |  | 830 (92%) | 73 (8%) | <0.001 |
|  | 2016-2020 |  | 315 (67%) | 155 (33%) |  |

Table S9: Significant explanatory variable “Italian region” associated with the infection by *A.* *phagocytophilum,* based on multivariate logistic regression; the numbers represent odds ratio of category on the row versus category on the column.

|  | Apulia | Calabria | Campania | Sardinia | Sicily | Abruzzo | Lazio | Marche | Tuscany | Umbria | Emilia-Romagna | Friuli-Venezia Giulia | Liguria | Lombardy | Piedmont | Trentino Alto-Adige | Vale d'Aosta | Veneto |
| --- | --- | --- | --- | --- | --- | --- | --- | --- | --- | --- | --- | --- | --- | --- | --- | --- | --- | --- |
| Apulia | - |  |  |  |  |  |  |  |  |  |  |  |  |  |  |  |  |  |
| Calabria | 3.72* | - |  |  |  |  |  |  |  |  |  |  |  |  |  |  |  |  |
| Campania | 0.91 | 0.24 | - |  |  |  |  |  |  |  |  |  |  |  |  |  |  |  |
| Sardinia | 2.95* | 0.79 | 3.25 | - |  |  |  |  |  |  |  |  |  |  |  |  |  |  |
| Sicily | 0.79 | 0.21* | 0.87 | 0.27** | - |  |  |  |  |  |  |  |  |  |  |  |  |  |
| Abruzzo | 3.81* | 1.02 | 4.20 | 1.29 | 4.82 | - |  |  |  |  |  |  |  |  |  |  |  |  |
| Lazio | 1.52 | 0.41 | 1.67 | 0.52 | 1.93* | 0.40 | - |  |  |  |  |  |  |  |  |  |  |  |
| Marche | 3.61* | 0.97 | 3.98 | 1.22 | 4.57* | 0.94 | 2.37 | - |  |  |  |  |  |  |  |  |  |  |
| Tuscany | 1.03 | 0.28* | 1.13 | 0.34** | 1.31 | 0.27 | 0.68 | 0.28* | - |  |  |  |  |  |  |  |  |  |
| Umbria | 4.73* | 1.27 | 5.21 | 1.60 | 5.98** | 1.24 | 3.10* | 1.31 | 4.58** | - |  |  |  |  |  |  |  |  |
| Emilia-Romagna | 1.05 | 0.28* | 1.15 | 0.36** | 1.32 | 0.28 | 0.69 | 0.29* | 1.02 | 0.22** | - |  |  |  |  |  |  |  |
| Friuli-Venezia Giulia | 1.07 | 0.29* | 1.19 | 0.36* | 1.36 | 0.28 | 0.71 | 0.30* | 1.04 | 0.22** | 1.02 | - |  |  |  |  |  |  |
| Liguria | 1.66 | 0.44 | 1.82 | 0.57 | 2.09* | 0.43 | 1.09 | 0.45 | 1.61 | 0.35 | 1.57 | 1.54 | - |  |  |  |  |  |
| Lombardy | 1.12 | 0.30* | 1.24 | 0.38* | 1.42 | 0.29 | 0.74 | 0.31* | 1.27 | 1.02 | 1.07 | 1.04 | 0.68 | - |  |  |  |  |
| Piedmont | 1.11 | 0.30* | 1.22 | 0.38* | 1.40 | 0.29 | 0.72 | 0.31* | 2.19* | 1.77 | 1.05 | 1.03 | 0.67 | 0.98 | - |  |  |  |
| Trentino Alto-Adige | 0.38* | 0.10** | 0.42 | 0.13*** | 0.48 | 0.10* | 0.25** | 0.11** | 1.63 | 1.32 | 0.37* | 0.36* | 0.23** | 0.34* | 0.35* | - |  |  |
| Aosta Valley | ne | ne | ne | ne | ne | ne | ne | ne | ne | ne | ne | ne | ne | ne | ne | ne | - |  |
| Veneto | 1.28 | 0.34 | 1.41 | 0.43* | 1.62 | 0.34 | 0.84 | 0.35* | 1.24 | 0.27* | 1.21 | 1.18 | 0.77 | 1.13 | 1.15 | 3.31** | ne | - |

* = *P* < 0.05; ** = *P* < 0.01; *** = *P* < 0.001; ne = not estimable.

***Ehrlichia canis***

Table S10. Characteristics of a cohort of 10334 dogs infected by *Ehrlichia canis*; data are expressed as n° of subjects (% on the total of each category). The frequency of categories for each variable was compared across affected and non-affected status by a *χ^2^* test, *P*<0.05 was considered significant.

| Variable | Category |  | No | Yes | *P*-value |
| --- | --- | --- | --- | --- | --- |
| Macro area | Northern Italy |  | 2962 (72%) | 1166 (28%) | <0.001 |
|  | Central Italy |  | 3170 (73%) | 1191 (27%) |  |
|  | Southern Italy |  | 1239 (67%) | 606 (33%) |  |
| Sex | Female |  | 3330 (70%) | 1420 (30%) | 0.090 |
|  | Male |  | 4041 (73%) | 1543 (27%) |  |
| Age | 0-5 years |  | 3651 (72%) | 1425 (28%) | 0.065 |
|  | 6-10 years |  | 2771 (71%) | 1122 (29%) |  |
|  | >10 years |  | 949 (69%) | 416 (31%) |  |
| Region | Apulia |  | 337 (70%) | 145 (30%) | <0.001 |
|  | Basilicata |  | 21 (55%) | 17 (45%) |  |
|  | Calabria |  | 157 (71%) | 65 (29%) |  |
|  | Campania |  | 91 (60%) | 61 (40%) |  |
|  | Molise |  | 5 (56%) | 4 (44%) |  |
|  | Sardinia |  | 173 (74%) | 61 (26%) |  |
|  | Sicily |  | 455 (64%) | 253 (36%) |  |
|  | Abruzzo |  | 106 (77%) | 38 (23%) |  |
|  | Lazio |  | 1287 (71%) | 523 (29%) |  |
|  | Marche |  | 441 (79%) | 115 (21%) |  |
|  | Tuscany |  | 1201 (72%) | 468 (28%) |  |
|  | Umbria |  | 135 (74%) | 47 (26%) |  |
|  | Emilia-Romagna |  | 675 (72%) | 268 (28%) |  |
|  | Friuli-Venezia Giulia |  | 212 (70%) | 93 (30%) |  |
|  | Liguria |  | 159 (67%) | 77 (33%) |  |
|  | Lombardy |  | 986 (74%) | 350 (26%) |  |
|  | Piedmont |  | 227 (71%) | 92 (29%) |  |
|  | Trentino Alto-Adige |  | 57 (65%) | 31 (35%) |  |
|  | Aosta Valley |  | 10 (53%) | 9 (47%) |  |
|  | Veneto |  | 636 (72%) | 246 (28%) |  |
| Year | 2006-2009 |  | 2224 (71%) | 923 (29%) | 0.004 |
|  | 2010-2015 |  | 3365 (72%) | 1288 (28%) |  |
|  | 2016-2020 |  | 1782 (68%) | 754 (32%) |  |

Table S11: Significant explanatory variable “Italian region” associated with the infection by *E. canis,* based on multivariate logistic regression; the numbers represent odds ratio of category on the row versus category on the column.

|  | Apulia | Basilicata | Calabria | Campania | Molise | Sardinia | Sicily | Abruzzo | Lazio | Marche | Tuscany | Umbria | Emilia-Romagna | Friuli-Venezia Giulia | Liguria | Lombardy | Piedmont | Trentino Alto-Adige | Vale d'Aosta | Veneto |
| --- | --- | --- | --- | --- | --- | --- | --- | --- | --- | --- | --- | --- | --- | --- | --- | --- | --- | --- | --- | --- |
| Apulia | - |  |  |  |  |  |  |  |  |  |  |  |  |  |  |  |  |  |  |  |
| Basilicata | 0.81 | - |  |  |  |  |  |  |  |  |  |  |  |  |  |  |  |  |  |  |
| Calabria | 1.69** | 2.09* | - |  |  |  |  |  |  |  |  |  |  |  |  |  |  |  |  |  |
| Campania | 1.03 | 1.27 | 0.61* | - |  |  |  |  |  |  |  |  |  |  |  |  |  |  |  |  |
| Molise | 0.90 | 1.12 | 0.54 | 0.88 | - |  |  |  |  |  |  |  |  |  |  |  |  |  |  |  |
| Sardinia | 1.99*** | 2.48* | 1.18 | 1.95*** | 2.21* | - |  |  |  |  |  |  |  |  |  |  |  |  |  |  |
| Sicily | 1.26* | 1.57 | 0.750 | 1.23 | 1.40 | 0.65** | - |  |  |  |  |  |  |  |  |  |  |  |  |  |
| Abruzzo | 2.38*** | 2.95** | 1.41 | 2.32** | 2.63*** | 1.19 | 1.87** | - |  |  |  |  |  |  |  |  |  |  |  |  |
| Lazio | 1.73*** | 2.14* | 1.03 | 1.69** | 1.91* | 0.86 | 1.36*** | 0.72 | - |  |  |  |  |  |  |  |  |  |  |  |
| Marche | 2.67*** | 3.32*** | 1.59* | 2.60*** | 2.96** | 1.33 | 2.11*** | 1.12 | 1.55*** | - |  |  |  |  |  |  |  |  |  |  |
| Tuscany | 1.81*** | 2.24* | 1.07 | 1.76** | 2.00* | 0.90 | 1.43*** | 0.76 | 1.04 | 0.68*** | - |  |  |  |  |  |  |  |  |  |
| Umbria | 2.01*** | 2.49* | 1.19 | 1.96** | 2.22* | 1.01 | 1.58* | 0.84 | 1.16 | 0.75 | 1.11 | - |  |  |  |  |  |  |  |  |
| Emilia-Romagna | 1.76*** | 2.18* | 1.04 | 1.71** | 1.94* | 0.88 | 1.39** | 0.67 | 1.01 | 0.65*** | 0.97 | 0.87 | - |  |  |  |  |  |  |  |
| Friuli-Venezia Giulia | 1.61*** | 2.00* | 0.96 | 1.57* | 1.78* | 0.80 | 1.27 | 0.68 | 0.93 | 0.60 | 0.89 | 0.8 | 0.92 | - |  |  |  |  |  |  |
| Liguria | 1.41* | 1.75* | 0.83 | 1.37 | 1.56 | 0.71 | 1.11 | 0.59* | 0.81 | 0.53*** | 0.78 | 0.70 | 0.81 | 0.88 | - |  |  |  |  |  |
| Lombardy | 1.96*** | 2.43** | 1.16 | 1.91*** | 2.17* | 0.98 | 1.54*** | 0.82 | 1.13 | 0.73* | 1.08 | 0.98 | 1.12 | 1.21 | 1.39* | - |  |  |  |  |
| Piedmont | 1.72*** | 2.14* | 1.02 | 1.68* | 1.90* | 0.86 | 1.36* | 0.72 | 0.99 | 0.64*** | 0.95 | 0.86 | 0.98 | 1.07 | 1.22 | 0.88 | - |  |  |  |
| Trentino Alto-Adige | 1.29 | 1.59 | 0.76 | 1.25 | 1.42 | 0.64 | 1.01 | 0.54* | 0.74 | 0.48** | 0.71 | 0.64 | 0.73 | 0.79 | 0.91 | 0.66 | 0.75 | - |  |  |
| Aosta Valley | 0.80 | 1.00 | 0.48 | 0.78 | 0.89 | 0.40* | 0.63 | 0.33* | 0.46 | 0.30* | 0.44* | 0.40* | 0.46 | 0.50 | 0.57 | 0.41* | 0.47 | 0.62 | - |  |
| Veneto | 1.84*** | 2.29* | 1.09 | 1.80** | 2.04* | 0.92 | 1.46*** | 0.77 | 1.06 | 0.69** | 1.02 | 0.92 | 1.05 | 1.14 | 1.30 | 0.94 | 1.07 | 1.43 | 0.43* | - |

* = *P* < 0.05; ** = *P* < 0.01; *** = *P* < 0.001; ne = not estimable.

Table S12: Significant explanatory variable “year” associated with the infection by *E. canis,* based on multivariate logistic regression; the numbers represent odds ratio of category on the row versus category on the column.

|  | 2006-2009 | 2010-2015 | 2016-2020 |
| --- | --- | --- | --- |
| 2006-2009 | - |  |  |
| 2010-2015 | 1.11 | - |  |
| 2016-2020 | 0.93* | 0.84** | - |

* = *P* < 0.05; ** = *P* < 0.01
